# Familial severe skeletal Class II malocclusion with gingival hyperplasia caused by a complex structural rearrangement at the *KCNJ2-KCNJ16 locus*

**DOI:** 10.1016/j.xhgg.2024.100352

**Published:** 2024-09-10

**Authors:** Reza Maroofian, Alistair T. Pagnamenta, Alireza Navabazam, Ron Schwessinger, Hannah E. Roberts, Maria Lopopolo, Mohammadreza Dehghani, Mohammad Yahya Vahidi Mehrjardi, Alireza Haerian, Mojtaba Soltanianzadeh, Mohammad Hadi Noori Kooshki, Samantha J.L. Knight, Kerry A. Miller, Simon J. McGowan, Nicolas Chatron, Andrew T. Timberlake, Uirá Souto Melo, Stefan Mundlos, David Buck, Stephen R.F. Twigg, Jenny C. Taylor, Andrew O.M. Wilkie, Eduardo Calpena

**Affiliations:** 1 Department of Neuromuscular Diseases, UCL Queen Square Institute of Neurology, London, UK; 2 National Institute for Health Research Oxford Biomedical Research Centre, Oxford, UK; 3 Wellcome Centre for Human Genetics, University of Oxford, Oxford, UK; 4 Faculty of Dentistry, Shahid Sadoughi University of Medical Sciences, Yazd, Iran; 5 Centre for Computational Biology, MRC Weatherall Institute of Molecular Medicine, University of Oxford, Oxford, UK; 6 Abortion Research Center, Yazd Reproductive Sciences Institute, Shahid Sadoughi University of Medical Sciences, Yazd, Iran; 7 Diabetes Research Center, Shahid Sadoughi University of Medical Sciences, Yazd, Iran; 8 Clinical Genetics Group, MRC Weatherall Institute of Molecular Medicine, University of Oxford, Oxford, UK; 9 Computational Biology Research Group, MRC Weatherall Institute of Molecular Medicine, University of Oxford, Oxford, UK; 10 Service de Génétique, Hospices Civils de Lyon, Lyon, France; 11 Hansjörg Wyss Department of Plastic Surgery, NYU Langone Medical Center, New York, NY, USA; 12 Max Planck Institute for Molecular Genetics, Berlin, Germany; 13 Grupo de Investigación en Biomedicina Molecular, Celular y Genómica, Unidad CIBERER, Instituto de Investigación Sanitaria La Fe (IIS La Fe), Valencia, Spain

**Keywords:** malocclusion, mandibular retrognathism, maxillary protrusion, maxillary prognathism, gingival hyperplasia, chromothripsis, chromoanagenesis, *KCNJ2*, *KCNJ16*, neo-TAD, enhancer hijacking

## Abstract

The aim of this work was to identify the underlying genetic cause in a four-generation family segregating an unusual phenotype comprising a severe form of skeletal Class II malocclusion with gingival hyperplasia. SNP array identified a copy number gain on chromosome 1 (chr1); however, this chromosomal region did not segregate correctly in the extended family. Exome sequencing also failed to identify a candidate causative variant but highlighted co-segregating genetic markers on chr17 and chr19. Short- and long-read genome sequencing allowed us to pinpoint and characterize at nucleotide-level resolution a chromothripsis-like complex rearrangement (CR) inserted into the chr17 co-segregating region at the *KCNJ2-SOX9 locus*. The CR involved the gain of five different regions from chr1 that are shuffled, chained, and inserted as a single block (∼828 kb) at chr17q24.3. The inserted sequences contain craniofacial enhancers that are predicted to interact with *KCNJ2/KCNJ16* through neo-topologically associating domain (TAD) formation to induce ectopic activation. Our findings suggest that the CR inserted at chr17q24.3 is the cause of the severe skeletal Class II malocclusion with gingival hyperplasia in this family and expands the panoply of phenotypes linked to variation at the *KCNJ2-SOX9 locus*. In addition, we highlight a previously overlooked potential role for misregulation of the *KCNJ2/KCNJ16* genes in the pathomechanism of gingival hyperplasia associated with deletions and other rearrangements of the 17q24.2-q24.3 region (MIM 135400).

## Teaser

This study identifies a complex chromosomal rearrangement involving chr1 insertion into chr17 as the genetic cause of severe skeletal Class II malocclusion with gingival hyperplasia in a four-generation family, revealing new insights into the role of *KCNJ2/KCNJ16* misregulation in craniofacial disorders.

## Introduction

The discovery of novel monogenic causes of rare disorders has greatly accelerated since the introduction of next-generation DNA sequencing (NGS) technologies; however, outside the coding regions, identifying and interpreting pathogenic implications of sequence changes remains challenging.^1^^,^^2^ This is particularly problematic for more complex genetic lesions such as copy number variants (CNVs) and structural variants (SVs). Such variants have the potential to disrupt the integrity of the genome, causing changes in the regulatory architecture that lead to pathogenic alterations of gene expression levels and/or patterns.^3^^,^^4^

Here, we describe a large family with an unusual phenotype presenting with gingival hyperplasia and a severe form of skeletal Class II malocclusion characterized by mandibular retrognathism with individual variations in maxillary positioning, including maxillary protrusion and extreme overjet in the proband. Hereditary gingival hyperplasia (also known as gingival fibromatosis) is a rare condition that may occur as an isolated trait or associated with several syndromes. To date, five distinct *loci* related to non-syndromic hereditary gingival hyperplasia have been identified (on chr2 [two *loci*], chr4, chr5, and chr11), and of these, only pathogenic variants in the *SOS1* (chr2) and *REST* (chr4) genes have been described.^5^ Skeletal malocclusion is a common defect that occurs due to abnormality of maxillary and/or mandibular development; however, severe maxillary prognathism is an unusual entity even in known craniofacial syndromes. Extensive bibliographic searches identified a report describing maxillary protrusion in several affected individuals (six out of seven) of a large consanguineous family; however, as a part of a more complex clinical picture (including severe intellectual disability and strabismus), with autosomal recessive inheritance and caused by a homozygous stop-gain at the *SOBP* gene.^6^^,^^7^ In addition, there are only a few reports describing cases presenting with a dominantly inherited non-syndromic gingival hyperplasia and severe skeletal Class II malocclusion,^8^ and to our knowledge, our work represents the first characterization of a fully penetrant genetic/genomic alteration associated with this phenotype in humans.

Using genome sequencing (GS) we identified and characterized at the nucleotide level a chromothripsis-like complex rearrangement (CR) involving chr1 inserted into a co-segregating region at the *KCNJ2-SOX9 locus* (chr17q24.3). Our findings suggest that the CR inserted at the topologically associating domain (TAD) containing the two potassium channel genes *KCNJ2* and *KCNJ16* cause the severe skeletal Class II malocclusion with gingival hyperplasia in the family, and that the most probable pathogenic mechanism underlying the abnormal phenotype involves *KCNJ2/KCNJ16* misregulation induced by neo-TAD formation and adopted craniofacial enhancers.

## Material and methods

### Patients

The study was approved by Ethics Committee of Shahid Sadoughi University of Medical Science of Yazd, Iran (Project 271653, IR.SSU.REC.1394.231) and informed written consent was obtained from all the subjects participating in the study. Permission was obtained to publish patient photographs or images. DNA was obtained from peripheral blood samples by phenol-chloroform extraction.

### Genetic analysis

Genomic DNA (200 ng) was analyzed using a 300K Human CytoSNP-12 BeadChip according to manufacturer’s guidelines (Illumina Inc). Data were processed using GenomeStudio (Illumina Inc) and analyzed using Nexus Discovery Edition v9 (BioDiscovery). Exome capture of DNA from patients was carried out using SureSelect Human All Exon Kit v6 (Agilent) following the manufacturer’s instructions. Libraries were prepared using 200 ng (ES) or 3 μg (GS) of genomic DNA extracted from whole blood; sequencing was performed on an Illumina HiSeq4000, with 75 or 151 base pair (bp) paired-end reads for ES or GS, respectively, and bioinformatic analysis of small variants (single nucleotide variant [SNV] and indels) in the exonic regions was performed as previously described.^9^ Analysis of GS-identified variants located outside the exons was conducted with the Ingenuity Variant Analysis (IVA; Qiagen) software. CNV analysis of GS data consisted of a combination of custom scripts, ngCGH (https://github.com/seandavi/ngCGH), and Nexus Discovery Edition v9 software (Biodiscovery).^10^ Complementary bioinformatic analysis to detect CNVs and SVs was implemented as recently described.^11^ Long-read GS was performed on PromethION (Oxford Nanopore Technologies) using libraries prepared with PCR-based or PCR-free, fragmentation-free methods as previously described.^10^ Mapping was done with minimap2 v 2.10 whereas structural variant calling employed Sniffles v1.0.9 with parameters -s 10, -l 100. Integrative Genomics Viewer (IGV) was used to visualize NGS data and to characterize the SVs. Confirmation and segregation analysis of variants was carried out by dideoxy-sequencing and/or restriction digest of genomic PCR amplification products.

### Genomic interpretation

Genomic features from the hg19 human genome build were downloaded from the UCSC genome browser. Coordinates from 4,399 mouse craniofacial enhancers,^12^ defined as regions that showed significant p300 binding in craniofacial tissue (mouse embryonic day 11.5 facial tissue) and were at least 2.5 kb from known transcription start sites, were downloaded and mapped to hg19 coordinates using the UCSC liftover tool. Human craniofacial enhancers,^13^ revealed by chromatin state segmentations from human embryonic craniofacial tissues, were visualized using the Human Craniofacial Epigenomics Hub available in the UCSC genome browser. Hi-C chromatin structure on IMR90^14^ (VC_SQRT normalization and 5 kb resolution, except 10 kb resolution in Figure S8) and CTCF binding sites (GSM935404 from the ENCODE project) were visualized in the UCSC genome browser. RNA sequencing (RNA-seq) from e11.5 mouse face subregions (medial-nasal, lateral-nasal, mandibular, and maxillary processes) from FaceBase (dataset TMJ)^15^ were visualized using the FaceBase Hub available in the UCSC genome browser (mm10), and the TPM (transcripts per million) information was extracted from FaceBase.

### Prediction of chromatin interactions

Chromatin interactions were predicted using deepC.^16^ To that end, a deepC model was trained on IMR90 Hi-C data.^14^ Contact matrices at 5 kb resolution were obtained from GEO (GSE63525). The model was trained following the architecture and procedure described in deepC, holding out chromosomes 12 and 13 for testing and 16 and 17 for validation. Chromatin interactions were then predicted based on DNA sequence inputs (1 Mb inputs in a 5 kb sliding window) from reference and the *in silico* produced CR-containing equivalent variant.

## Results

**Figure 1:**
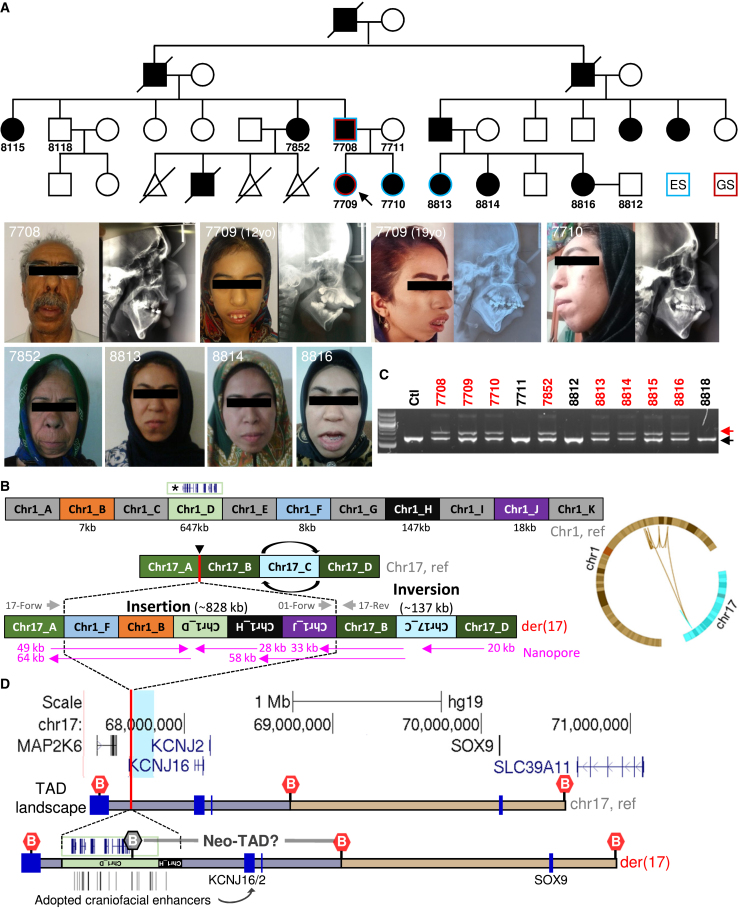
Pedigree, genomic rearrangement, and hypothetical mechanism (A) Pedigree and clinical photographs of the family. Circles represent females, squares represent males, and triangles represent miscarriages. Filled symbols represent affected individuals, black arrow depicts the proband, and individuals with DNA available are shown with their sample ID below the symbol. Symbols with borders in red and/or blue indicate the family members investigated by genome and/or exome sequencing, respectively. At the bottom, clinical pictures and lateral radiographs are shown for available family members. (B) Schematic diagram showing the reference regions in chr1 and chr17 and the derivative chr17. The five duplicated regions from chr1 are shown in different colors whereas the non-duplicated regions are shown in gray. Although the segments are not scaled, the representation shows the relative positions and orientations, and the sizes of the segments involved in the rearrangement are shown. The five protein-coding genes (*MROH9*, *FMO3*, *FMO2*, *FMO1*, and *FMO4*) included in the chr1_D region are represented at the top of the chr1_D segment and are indicated with an asterisk. The insertion point in chr17 is indicated with a black arrowhead and a vertical red line, and the concomitant inversion of the light blue segment in the chr17 is indicated by clockwise arrows. The relative position of the primers used for the segregation analysis shown in (C) are represented as gray arrows in the diagram of the derivative chr17, and below the diagram, the approximate positions of 20–64 kb nanopore long reads, including four which span multiple breakpoints, are shown in pink. On the right is shown a circos plot visualization of the rearrangement identified in the family. (C) Segregation analysis using a duplex PCR assay based on three primers to detect the mutant allele (upper fragment; product from chr1-forward and chr17-reverse primers) and the normal allele (lower fragment; product from chr17-forward and chr17-reverse primers). Sample IDs are shown at the top of the agarose gel with the affected individuals in red color, and “ctl” indicates a genomic sample from a control individual unrelated to this family. (D) UCSC track showing the coordinates (hg19) and genes in the region of interest in the chr17. The insertion site of the duplicated regions from chr1 is indicated by the red line, whereas the concomitant inversion in the chr17 is shown in blue highlight. At the bottom is shown a scaled representation of the TAD landscape based on Franke et al.,^17^ showing the genes as blue boxes, the TAD boundaries as hexagonal symbols, and below is represented the theoretical prediction of the TAD landscape in the derivative chr17. For simplicity, only the two larger segments (chr1_D and chr1_H) containing human craniofacial enhancers are depicted. Note that the five protein-coding genes (*FMO4*, *FMO1*, *FMO2*, *FMO3*, and *MROH9*) represented at the top of the inserted chr1_D are positioned upstream of the TAD boundary (which is also included in chr1_D) and are not part of the neo-TAD containing the *KCNJ2/KCNJ16* genes.

We investigated a large Iranian family affected with severe skeletal Class II malocclusion and gingival hyperplasia that segregated through four generations (Figure 1A). The phenotype of the nuclear family (IDs 7708, 7709, 7710) is summarized in Table 1. Although the family was initially identified as having maxillary prognathism based on their clinical presentation alone, cephalometric analyses suggest a more intricate situation. Some affected family members have true maxillary protrusion, while others exhibit a similar phenotype without the characteristic cephalometric measurements of the condition. The cephalometric data reveal a severe skeletal Class II malocclusion (A point to B point angle, ANB 9°–10°) characterized by mandibular retrognathism (Sella-Nasion to B point angle, SNB <78°) with individual variations in maxillary positioning, including maxillary protrusion (Sella-Nasion to A point angle, SNA >84°) and extreme overjet (11 mm) in the proband, contrasted with a retrusive maxilla in the father (SNA <80°). Additionally, they show an increased mandibular plane angle (42°–49°). In order to identify a presumed genetic cause inherited as a fully penetrant autosomal dominant trait (X-linked transmission was excluded by the presence of male-to-male transmission), exome sequencing (ES) was performed using genomic DNA from four initially available affected individuals (first the father/proband/sister and subsequently a second cousin; IDs 7708, 7709, 7710, and 8813, respectively; Figure 1A). Additional segregation analysis was performed using DNA obtained from four further affected individuals (7852, 8814–8816), one unaffected individual at 50% prior risk (8118), and two spouses (7711, 8812; Figure 1A). Initial investigation of 30 novel/rare variants identified on ES (selected by deleteriousness or the biological relevance of affected genes) failed to identify any fully co-segregating candidate (Table S1). Instead, additional analysis (including more frequently observed variants) of the ES data allowed us to determine fully co-segregating genetic markers from chr17 and chr19 (Table S1); no pathogenic variants were detected by ES in these two regions.

**Table 1:** Summary of the main phenotype of the nuclear family

ID	7709 (proband)	7710 (sister)	7708 (father)
Gender	Female	Female	Male
Age at exam (years)	19 (previous visits at 5, 12, 17)	25	54
Occlusion (Angle classification)	Class II	Class II	Class II
SNA (degrees); normal range 80-84^a^	85	81	75
SNB (degrees); normal range 78-82^a^	75	72	65
ANB (degrees); normal range 1-5^a^	10	9	10
Maxillary length (mm). McNamara’s method	47	71	71
Mandibular length (mm). McNamara’s method	63	107	117
Mandibular plane (degrees). Frankfort Horizontal plane. Mean reference value 32^a^	42	52	79
Overjet (mm); ideal range 1-2^b^	11	3	N/A
Overbite (mm); ideal range 0-2^b^	4	1	N/A
Orthognathic procedures	None	None	None
Dental anomalies		Impacted upper 8s	
Gingival hyperplasia	Gingivectomy and gingivoplasty performed at age 5 years to uncover all deciduous teeth. Gingivoplasty performed at age 12 years for permanent teeth uncoverage	Gingivoplasty performed at age 9 years for teeth uncoverage	Present No surgery
Hypertrichosis^c^	N/A	N/A	N/A
Other congenital anomalies	–	–	–

SNA, Sella-Nasion to A point angle; SNB, Sella-Nasion to B point angle; ANB, A point to B point angle; N/A, not available.

a Reference values from Jacobson et al.^18^

b Reference values from Proffit et al.^19^

c Hypertrichosis has not been formally evaluated by a specialist.

SNP array analysis performed on the proband’s DNA did not identify any pathogenic CNV(s) within either of the two co-segregating candidate regions (chr17 and chr19), but revealed a heterozygous ∼645 kb duplication of chr1 downstream of the *PRRX1* gene (Figure S1), a known developmental disease gene involved in the agnathia-otocephaly syndrome (MIM 202650) and craniosynostosis.^20^^,^^21^ However, the microsatellite marker *D1S2815*, located ∼300 kb downstream of the duplicated region did not co-segregate with the phenotype in the family (Figure S1).

To investigate these genomic clues further, we undertook short-read GS of samples from the proband and her father. No potentially pathogenic small variants were evident in the two co-segregating candidate regions (chr17 and chr19), either in coding or non-coding sequences. CNV analysis of the GS data confirmed the previously identified duplication within chr1 but detected further complexity with additional smaller duplicated regions along chr1 (Figure S1). IGV inspection of the breakpoints uncovered in total five different duplicated regions (fragments ranging 7–647 kb) from chr1 that appeared shuffled, chained, and inserted as a single block (∼828 kb) in the chr17 co-segregating region at the *KCNJ2-SOX9 locus* (Figures 1B and S2). A close examination of the GS data at this chr17 *locus* additionally revealed the presence of a ∼137 kb inversion (Figures 1B and S2) near the insertion site (∼19 kb downstream). This CR likely originated by a chromothripsis-like event (briefly described as individual number 10 from Chatron et al. and 007MAX001 in Pagnamenta et al.),^11^^,^^22^ fully co-segregates with the phenotype in the family, as demonstrated by PCR-based validations (Figures 1C and S3). Furthermore, PCR and dideoxy-sequencing analysis validated all the breakpoints (BPs) unraveled by short-read GS (Figure S3). Long-read nanopore-based GS corroborated the presence of all the identified BPs (Figure 1B) and importantly, no additional complexity was discovered in this or in any other region, including either the additionally linked region of chr19 or the unlinked region of chr1 from which the insertion fragments were ancestrally sourced.

The duplicated regions harbor a total of five protein-coding genes: *MROH9* and members of the flavin-containing monooxygenase family, including *FMO3* (mutations cause the recessive disorder Trimethylaminuria; MIM 602079), *FMO2*, *FMO1*, and *FMO4*, all located within the chr1_D region (Figures 1B, S4, and S5). In gnomAD (v4) there are at least three large duplications that also encompass these genes (variant identifiers DUP_CHR1_5720012A, 38985_DUP, and 38986_DUP), with the 38985_DUP variant identified in five different individuals (gnomAD total frequency 0.000011). In addition, none of the orthologous genes are expressed in embryonic mouse face subregions (Figure S6). Therefore, it is unlikely that the increased dosage of these genes alone could explain the observed phenotype in this family. The gained regions (from chr1), and the concomitant inversion, are located at the *KCNJ2-SOX9 locus* (chr17q24.3; Figure 1D). Considering the distribution of the TADs of this well-characterized region,^23^^,^^17^ most probably the regulation of *SOX9* is unaffected, as the SOX9 TAD is not directly perturbed by the CR (Figure 1D). However, the CR is inserted in the TAD containing the two potassium channel genes *KCNJ2* and *KCNJ16* (referred to hereafter as KCNJ2/16 TAD), and the two larger regions from chr1 (chr1_D and chr1_H in Figure 1, 647.4 and 146.8 kb, respectively) contain craniofacial enhancers (Figures 1D, S4, and S5)^12^^,^^13^ that would have the potential to interact with the *KCNJ2/KCNJ16 loci*. Although the activity of these enhancers has not been formally tested (e.g., using mouse *LacZ* transgenic assays), they were identified with enhancer-associated markers using chromatin immunoprecipitation-sequencing (ChIP-seq) in human or mouse embryonic craniofacial tissues.^12^^,^^13^

Both orthologous *Kcnj2* and *Kcnj16* genes are not (or very lowly) expressed in e11.5 mouse face subregions (Figure S6). However, genes near/flanking the equivalent duplicated regions containing the craniofacial enhancers (i.e., *Prrx1*, *Prrc2c* and *Ivns1abp*) are expressed at higher levels in these craniofacial regions (including the maxillary and mandibular processes; Figure S6). It is therefore possible that any of these enhancers (contained in the duplicated regions inserted at chr17q24.3) are able to drive expression in craniofacial territories. Misexpression of *KCNJ2* is the cause of Cooks syndrome (MIM 106995), a congenital limb malformation characterized by aplasia of nails and short digits.^17^ Thus, misexpression of *KCNJ2* in skeletal tissue can lead to growth and patterning defects. The largest region from chr1 (chr1_D) additionally contains a putative TAD boundary (Figures 1D, S4, and S5), which could result in the formation of a neo-TAD containing the *KCNJ2/KCNJ16* genes and adopted craniofacial enhancers included in the CR (Figure 1D), but excluding the five protein genes (located upstream of the boundary in the inserted chr1_D; Figure 1D). Unfortunately, it was not possible to obtain patient cells to test this hypothetical model or to functionally validate the effect of the CR on the regulation of *KCNJ2/KCNJ16* genes.

**Figure 2:**
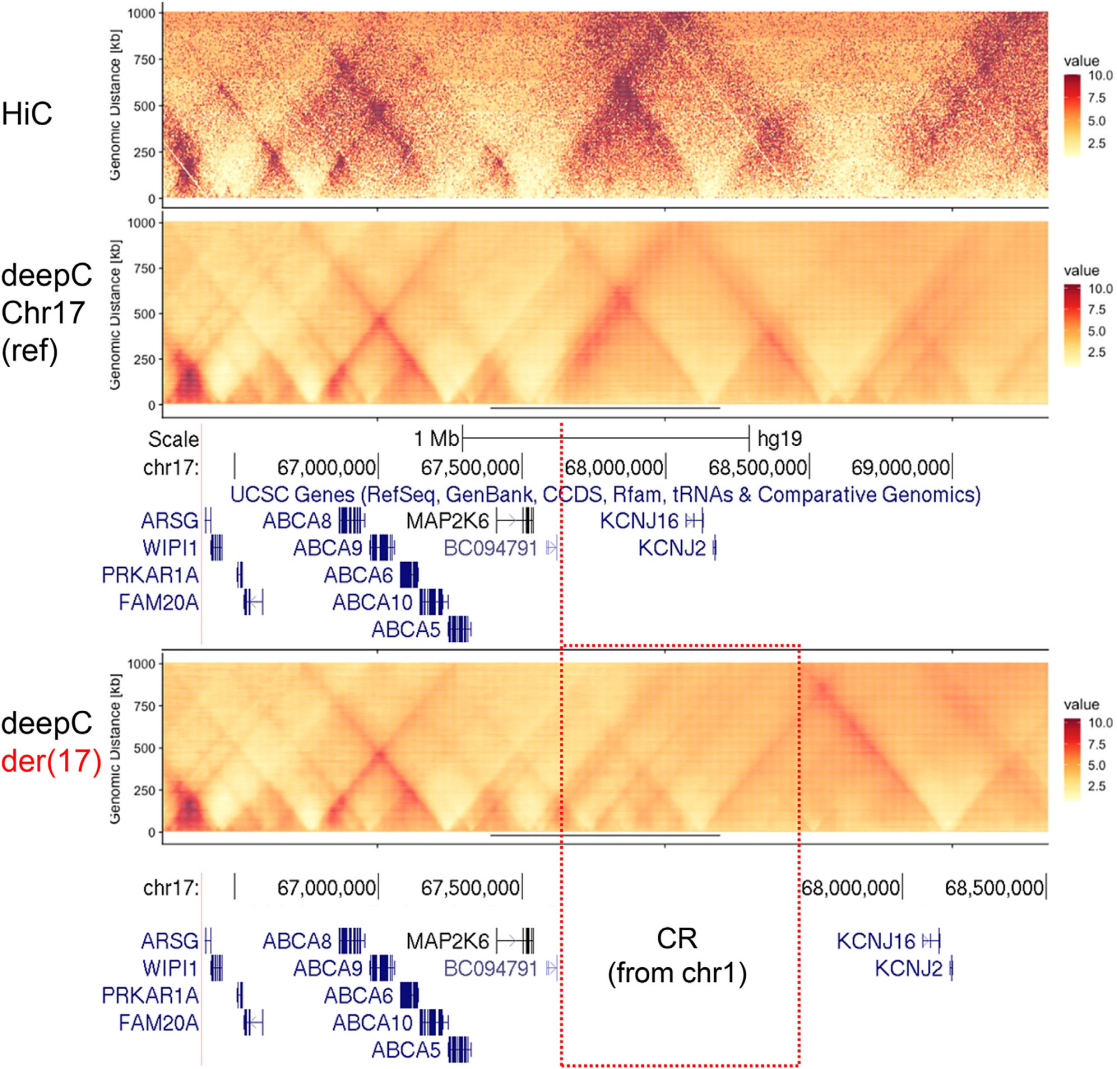
Hi-C data and deepC predictions at the *KCNJ2-KCNJ16 locus* On the top, the distance normalized Hi-C data of IMR90 cells is shown for the region of interest at chr17, whereas deepC predictions are shown for the reference sequence (middle panel) and for the CR-containing equivalent variant (lower panel), where the position of the inserted CR from chr1 is indicated. The color-coded values represent the interaction frequency in normalized Hi-C and predictions. UCSC tracks with the coordinates (hg19) and genes at the *KCNJ2-KCNJ16 locus* are shown below the predictions.

Alternatively, we used deepC, a recently described method developed using a transfer-learning-based deep neural network that accurately predicts chromatin interactions from DNA sequence,^16^ in order to add more insights into the impact produced by the insertion of the CR on the genome architecture at the *KCNJ2-KCNJ16 locus*. As shown in Figure 2, compared with the already available HiC data,^14^ deepC accurately predicted the landscape of genomic interactions at the *KCNJ2-KCNJ16 locus* in IMR90 cells (fibroblasts; same cell type previously used to investigate rearrangements at the *KCNJ2-SOX9 locus*).^24^ When computationally reproducing the CR (together with the concomitant inversion) in IMR90 cells, deepC predicted significant modifications in the genome architecture at the *KCNJ2-KCNJ16 locus* compared with the reference. Crucially, deepC predictions support the neo-TAD formation (Figure 2) induced by the presence of the TAD boundary incorporated from the largest chr1 fragment (chr1_D; Figure S5). The newly formed TAD contains the *KCNJ2/KCNJ16* genes, as well as craniofacial enhancers from chr1 (chr1_H and part of chr1_D). Of note, no major disturbances are observed when analyzing the 137 kb inversion alone, without the CR inserted in the *KCNJ2-KCNJ16 locus* or when comparing the effect of the CR with or without the inversion (Figure S7), likely excluding the inversion from having a major pathogenic role. As a whole, the deepC prediction supports our hypothesis and suggests that the most probable pathogenic mechanism underlying the severe skeletal malocclusion and gingival hyperplasia involves *KCNJ2/KCNJ16* misregulation induced by neo-TAD formation and adopted craniofacial enhancers.

## Discussion

The main purpose of this work was to delineate a genetic cause in a large family with an unusual phenotype presenting with a severe form of skeletal Class II malocclusion and gingival hyperplasia.

After thorough genetic investigations, we identified and characterized a CR inserted in the *KCNJ2*-*SOX9 locus*, which co-segregates with the phenotype in the family. Pathogenic variants at this *locus* (including CNVs and SVs) have been linked to various human diseases with a broad range of phenotypes, depending on the position, type of variant, extent of the affected region and effect on *KCNJ2-SOX9* expression/regulation.^3^^,^^17^^,^^25^ However, to our knowledge, severe skeletal Class II malocclusion caused by maxillary protrusion and/or mandibular retrusion has not been described as part of any of the known KCNJ2-SOX9-related phenotypes. Of note, the CR identified in the family is inserted into the KCNJ2/16 TAD, whereas the SOX9 TAD remains unperturbed and thus, it is very unlikely that *SOX9* regulation is affected.

Biallelic loss-of-function variants in *KCNJ16* cause renal tubulopathy associated with sensorineural deafness,^26^ whereas heterozygous variants in *KCNJ2* producing dominant-negative effects are responsible for Andersen-Tawil syndrome, characterized by periodic paralysis, cardiac arrhythmias, and dysmorphic features.^27^ Maxillary prognathism and gingival hyperplasia are not phenotypic features associated with these syndromes. However, the pathogenic mechanisms of previously described disease-causing variants in *KCNJ16* and *KCNJ2* differ from that proposed in this family.

Interestingly, in the literature there are a series of microdeletions centromeric to *KCNJ2/KCNJ16* that have been identified in individuals with congenital generalized hypertrichosis with or without gingival hyperplasia (microdeletion 17q24.2-q24.3 syndrome; MIM 135400; Figure S8). Although the hypertrichosis has been attributed to deletion of the *ABCA5* gene,^28^^,^^29^ the cause of the gingival hyperplasia observed in some of these patients is less clear. As these deletions include the TAD and boundaries located near *MAP2K6*, a misregulation effect on *KCNJ2/KCNJ16* is plausible. In line with this, there are at least two instances of complex SV with BPs near the *KCNJ2*/*KCNJ16* genes that potentially modify their regulatory landscape in individuals with syndromic gingival hyperplasia. An inverted duplication (tail-to-tail) of ∼1.5 Mb (encompassing the *ABCA8*, *ABCA9*, *ABCA6*, *ABCA10*, *ABCA5*, *MAP2K6* and *KCNJ2*/*KCNJ16* genes; Figure S8) was identified in a patient with extreme congenital generalized hypertrichosis terminalis, coarse face, and gingival hyperplasia.^30^ In addition, a reciprocal translocation t(3;17)(p14.3;q24.3), with the BP at 17q24.3 defined at the telomeric side of *KCNJ2* (Figure S8), was reported in a sporadic case with Zimmermann-Laband syndrome, presenting with gingival hyperplasia, hypertrichosis, unusually large ears and marked hypertrophy of the nose, and bulbous soft tissues of the fingertips.^31^^,^^32^ Of note, gain-of-function mutations in different potassium channels (*KCNN3*, *KCNH1*, and *KCNK4*) are responsible for syndromes (including Zimmermann-Laband) that share developmental delay and/or intellectual disability, coarse facial features, gingival enlargement, and hypertrichosis among other features.^33^ Altogether, a misregulation of the potassium channel *KCNJ2*/*KCNJ16* genes seems likely to contribute to the gingival hyperplasia. Although hypertrichosis is a relevant phenotype associated with this *locus*, we were unable to obtain a formal evaluation to confirm/assess its presence within the family. In general, family members have thick eyebrows and thick hairs, which is not limited to affected individuals and is likely attributed to their ethnicity.

The deepC predictions support the neo-TAD formation, likely inducing the ectopic expression of *KCNJ2/KCNJ16* by adopted craniofacial enhancers from chromosome 1. The larger inserted regions (chr1_D and chr1_H) contain craniofacial enhancers near/flanking genes highly expressed during the development of the mandibular and maxillary processes in mouse (i.e., *Prrx1*, *Prrc2c* and *Ivns1abp*; Figure S6), where *Kcnj2* and *Kcnj16* are not (or very lowly) expressed. From these, the *Prrx1* stands out as previous observations from human genetics and mouse models have demonstrated the importance of this gene for craniofacial development, including for jaw formation. In humans, *PRRX1* loss-of-function variants are linked to agnathia-otocephaly syndrome (MIM 202650), characterized by mandibular hypoplasia among other craniofacial defects, as well as craniosynostosis.^20^^,^^21^ In mice, the phenotype of the *Prrx1* homozygous knockout includes several defects in the development of both maxilla and mandible.^34^ The chr1_D contains a large genomic region with several craniofacial enhancers located just downstream of *PRRX1* (<14 kb from the last exon; Figure S5), which in the neo-TAD have the potential to interact with the *KCNJ2/KCNJ16* genes. We speculate that the ectopic activation of *KCNJ2/KCNJ16* in the jaw is responsible for the skeletal Class II malocclusion in the family. In line with this, it has already been shown that misexpression of *KCNJ2* in skeletal tissue can lead to growth and patterning defects. For example, Franke et al. demonstrated that *KCNJ2* misexpression is the cause of Cooks syndrome (MIM 106995), a congenital limb malformation characterized by aplasia of nails and short digits, as a consequence of CNVs producing neo-TAD formation (Figure S8).^17^

The main limitations of our report are that it is based on only one family and no functional data (for example, 3C-based methodologies and RNA-seq analyzing differentiated cells from patient-derived induced pluripotent stem cells) are available to demonstrate the hypothetical mechanism. We were unable to obtain patient cells from the family, and the size and complexity of the identified CR challenges the possibility of genetically engineering the exact variant. However, our work illustrates a generic approach to analyze and interpret complex SV by implementing deepC, a powerful tool able to accurately predict chromatin interactions from DNA sequence.

In summary, our findings suggest that the CR inserted at chr17q24.3 causes the skeletal Class II malocclusion with gingival hyperplasia in the family, and that the most probable pathogenic mechanism underlying the abnormal phenotype involves *KCNJ2/KCNJ16* misregulation induced by neo-TAD formation and adopted craniofacial enhancers. More broadly, we argue that *KCNJ2/KCNJ16* misregulation likely represents the common underlying mechanism in several previously reported patients with gingival hyperplasia and/or hypertrichosis, who harbor deletions and other rearrangements of the 17q24.2-q24.3 region (MIM 135400).

## Data and code availability

All the publicly available data used for the genomic interpretations and the prediction of chromatin interactions are indicated in the material and methods section. Exome or genome sequencing data obtained from patients are available upon request.

## Acknowledgments

We thank all the family members for their participation. This work was funded by the 10.13039/501100000272National Institute for Health Research (NIHR) Oxford Biomedical Research Centre (E.C., S.R.F.T., J.C.T., A.O.M.W.), 10.13039/100004440Wellcome (Senior Investigator Award 102731/Z/13/Z to A.O.M.W.), and MRC (Project Grant MR/T031670/1 to A.O.M.W.). This research was also funded and supported by the Wellcome Trust and Department of Health as part of the Health Innovation Challenge Fund scheme (R6-388 / WT100127) awarded to J.C.T. E.C. is supported by the Miguel Servet fellowship (CP23/00073) from 10.13039/501100004587Instituto de Salud Carlos III (ISCIII, Spain) and co-funded by the 10.13039/501100004895European Social Fund Plus (ESF+) from the European Union.

## Declaration of interests

The authors declare no competing interests.

## Supplemental information

Document S1. Figures S1–S8

Table S1. Co-segregation analysis

Document S2. Article plus supplemental information
