# Supplementary material for: Familial severe skeletal Class II malocclusion with gingival hyperplasia caused by a complex structural rearrangement at the KCNJ2-KCNJ16 locus
Source: HGG Adv. 2024 Sep 10;5(4):100352. doi: 10.1016/j.xhgg.2024.100352 (PMC11465088; doi:10.1016/j.xhgg.2024.100352)
Supplement: Document S1. Figures S1–S8 [file mmc1.pdf]

HGGA, Volume *5*

## Supplemental information

# Familial severe skeletal Class II malocclusion with gingival hyperplasia caused by a complex structural rearrangement at the *KCNJ2-KCNJ16 locus*

**Reza Maroofian, Alistair T. Pagnamenta, Alireza Navabazam, Ron Schwessinger, Hannah E. Roberts, Maria Lopopolo, Mohammadreza Dehghani, Mohammad Yahya Vahidi Mehrjardi, Alireza Haerian, Mojtaba Soltanianzadeh, Mohammad Hadi Noori Kooshki, Samantha J.L. Knight, Kerry A. Miller, Simon J. McGowan, Nicolas Chatron, Andrew T. Timberlake, Uirá Souto Melo, Stefan Mundlos, David Buck, Stephen R.F. Twigg, Jenny C. Taylor, Andrew O.M. Wilkie, and Eduardo Calpena**

**Fig. S1**

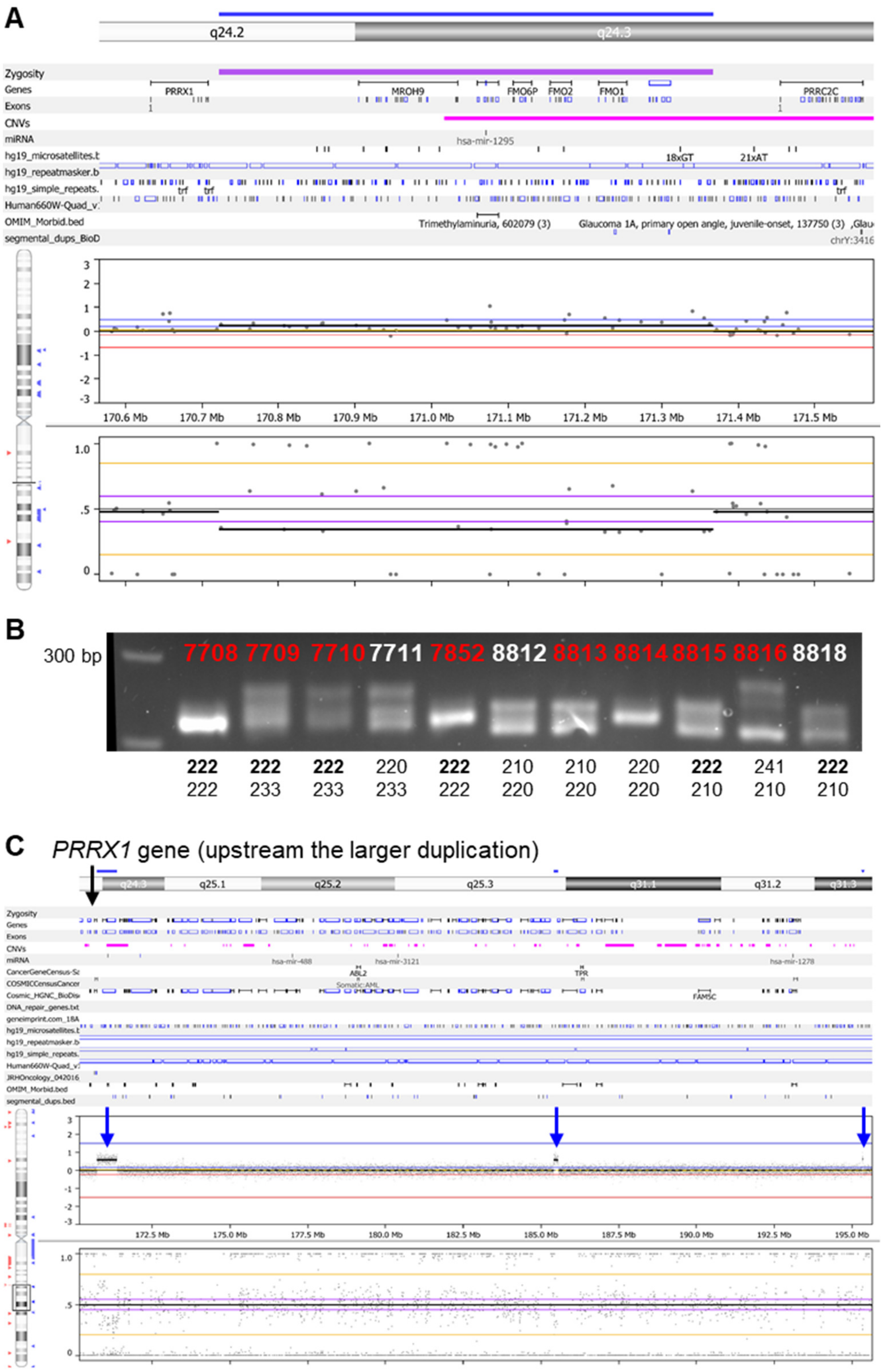


**Fig. S1.** (A) SNP array data at chr1 visualized using the Nexus software, showing on top the normalized signal intensity data (log R ratio, LRR) and the B-allele frequency (BAF) at the bottom. (B) Segregation analysis of the microsatellite marker *D1S2815*. Sample IDs are shown at the top of the gel with the affected individuals in red colour, and at the bottom the approximate fragment sizes obtained from the analysis in an Applied Biosystems 3730 Genetic Analyzer. (C) CNV analysis of GS data at chr1 visualized using the Nexus software, showing on top the normalized dosage and the BAF at the bottom.

Fig. S2

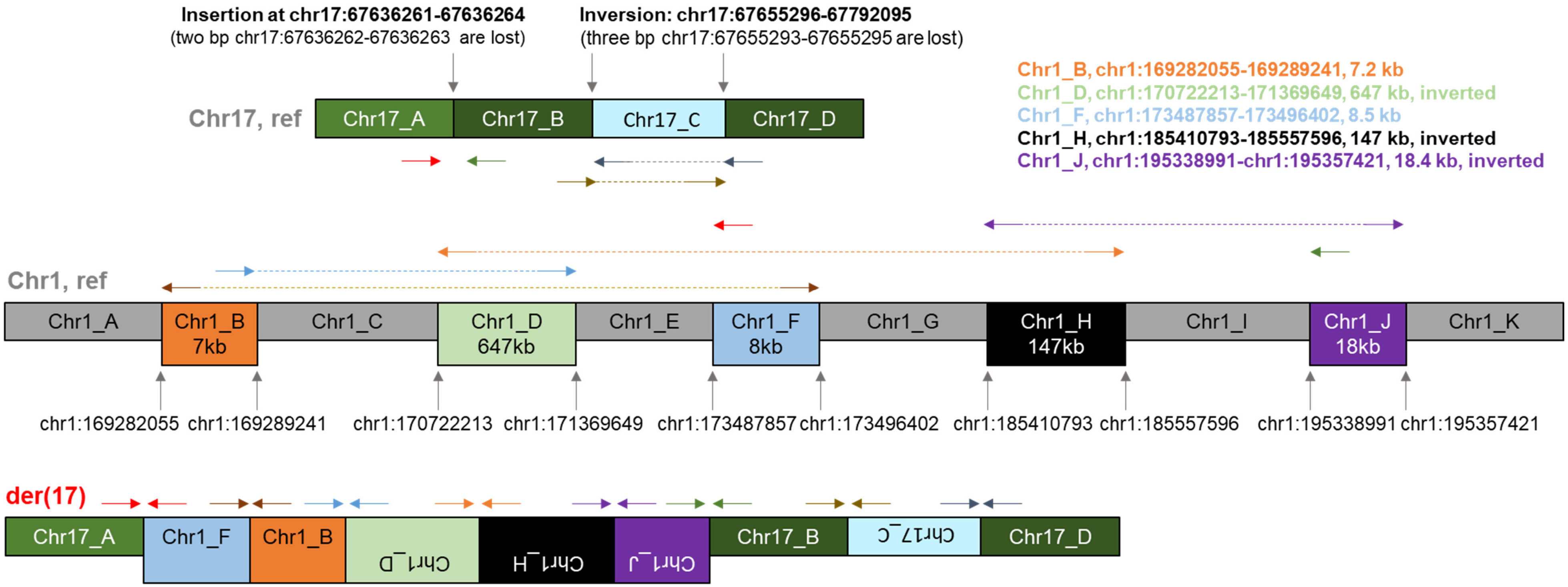


**Fig. S2.** Schematic diagram of the short-read GS data supporting the CR identified in the family. Representation of the reference regions in chr1 and chr17 and the derivative chr17 showing informative reads from short-read GS data to visualize the detection of the CR identified in the family. Genomic regions with increased dosage (detected by CNV analysis) are shown as taller boxes. Although the segments are not scaled, the representation shows the relative positions and orientations, and the sizes of the segments involved in the rearrangement are shown. Genomic coordinates are based on hg19.

**Fig. S3**

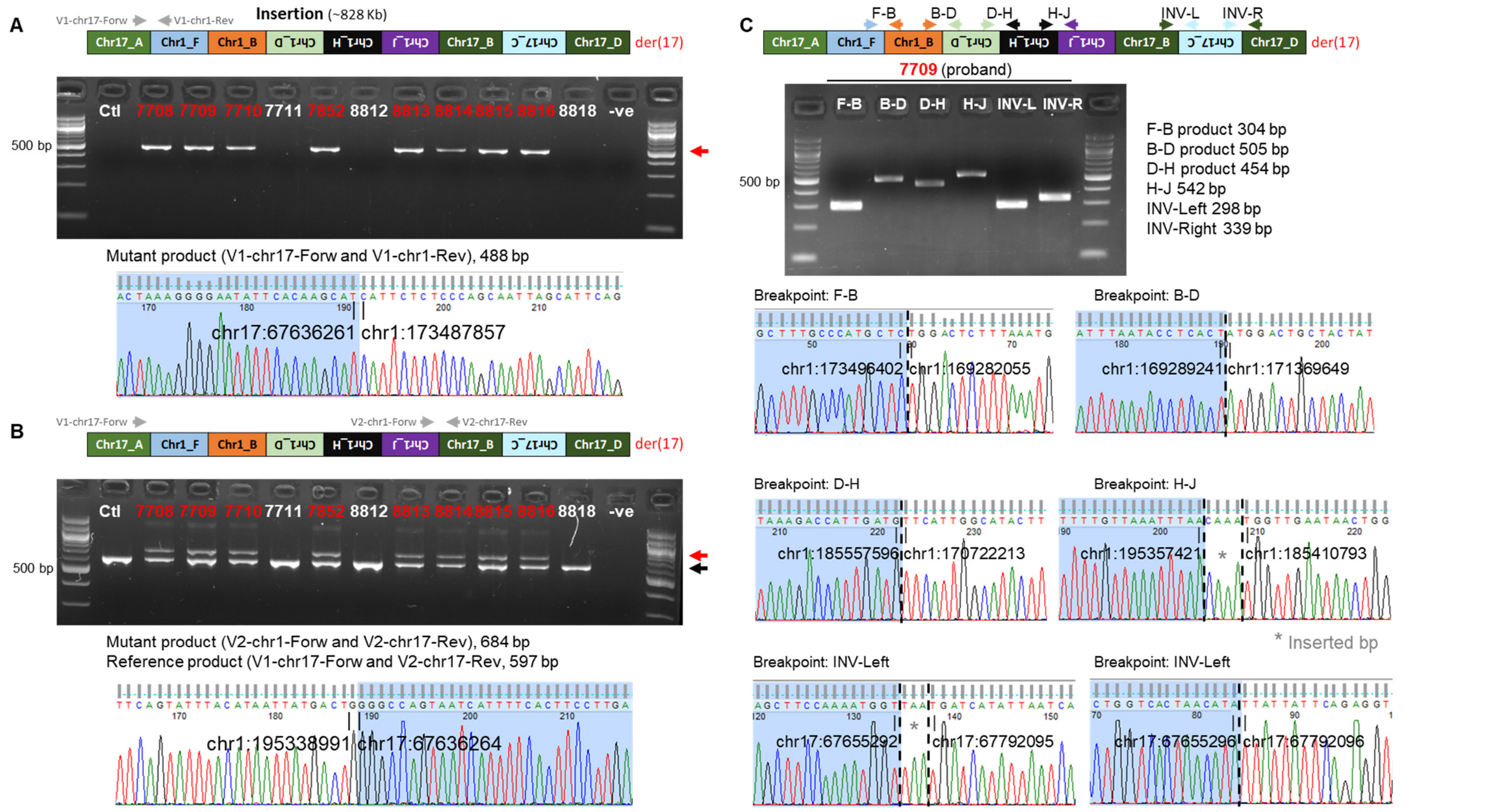


**Fig. S3.** PCR validation and dideoxy-sequencing of all the breakpoints (BPs). Segregation analysis of the CR identified in the family using BP-PCR at the chr17-chr1 (A) and chr1-chr17 (B). PCR validation of all the additional BPs are show in C, including the inversion. Note that for the inversion, 3 bp (chr17:67655293-67655295) are lost from the start of Chr17_C and 3 bp (chr17:676552096-676552098, TTA) are present at both inversion junctions.

**Fig. S4**

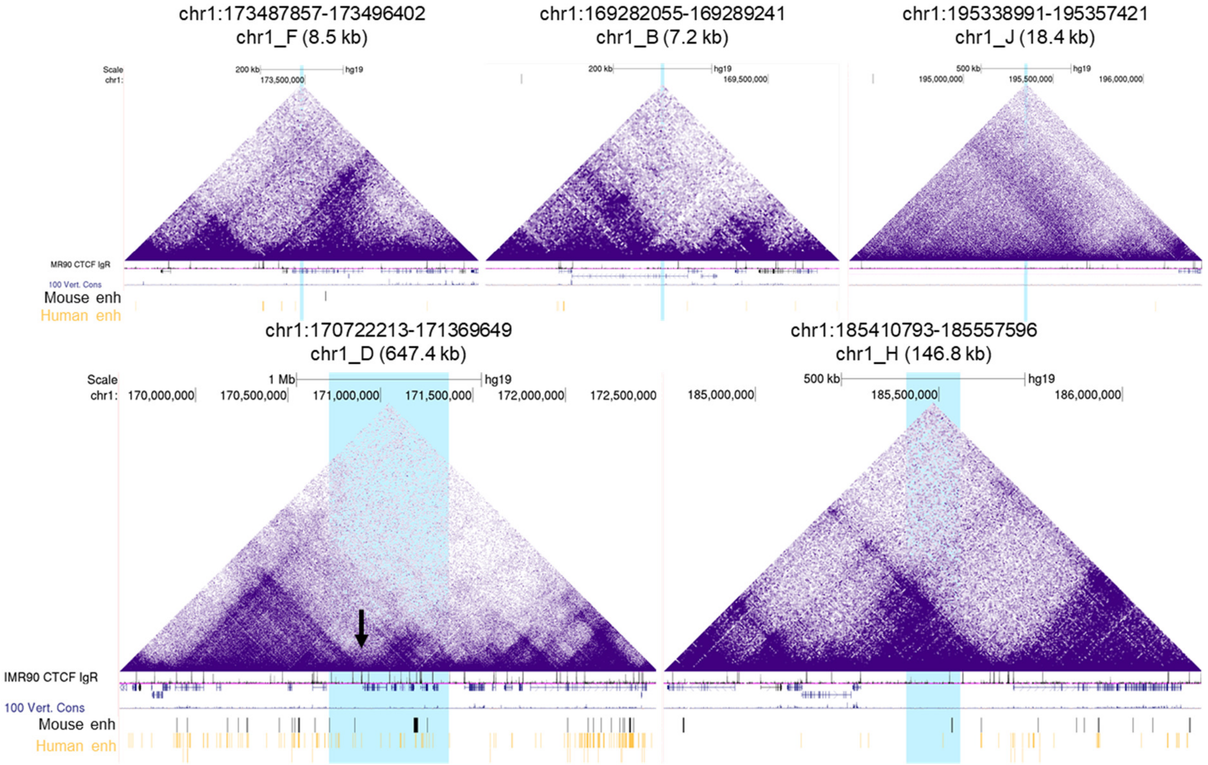


**Fig. S4.** TAD landscape and craniofacial enhancers at the regions from chr1. Individual panels show each of the five different regions from chr1 (highlighted in light blue) that are duplicated and inserted in the CR. In each panel, UCSC tracks depict Hi-C data at the top, CTCF ChIP-seq data, genes, and the craniofacial enhancers at the bottom. The black arrow indicates a TAD boundary included in the chr1_D duplicated region.

**Fig. S5**

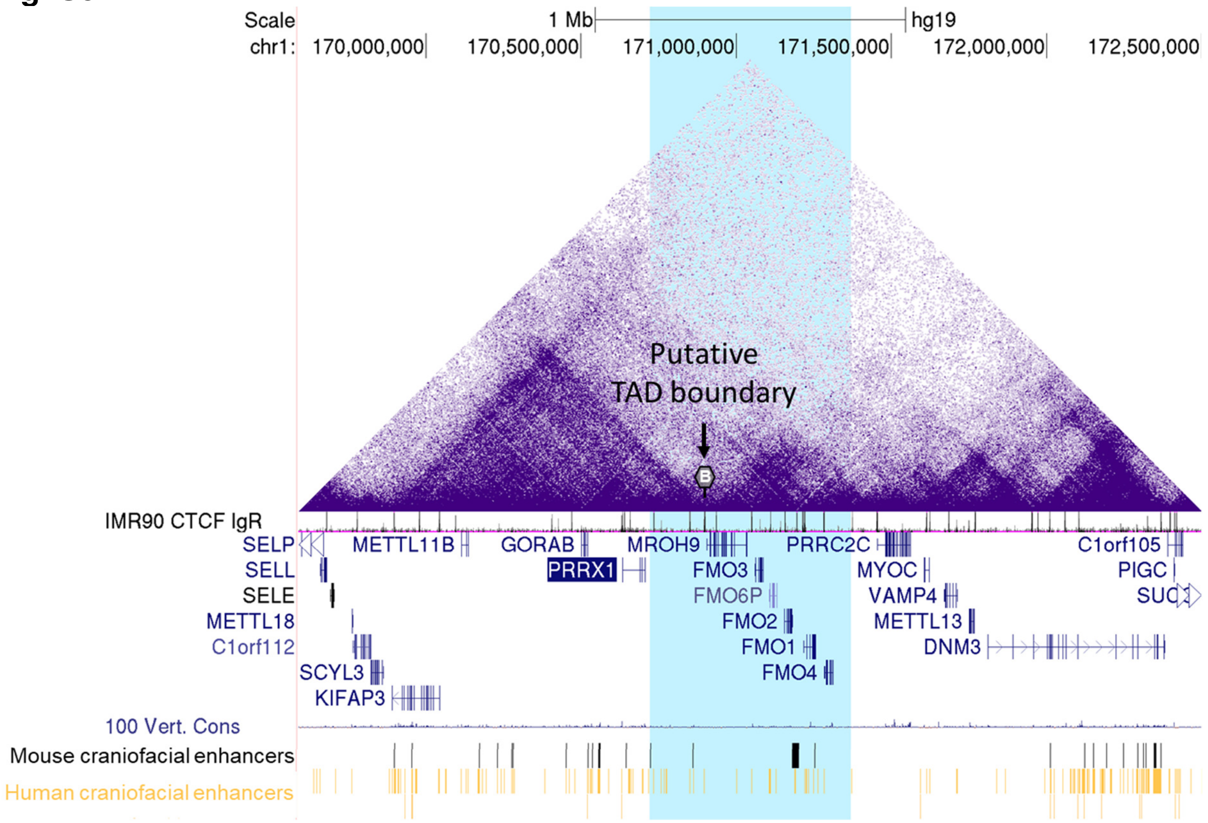


**Fig. S5.** TAD landscape and craniofacial enhancers at the human *PRRX1* region. Detailed panel of the chr1_D region (highlighted in light blue), showing UCSC tracks with Hi-C data at the top, CTCF ChIP-seq data, gene names and the craniofacial enhancers at the bottom. The black arrow indicates a TAD boundary (labelled B) included in the chr1_D region.

## Fig. S6

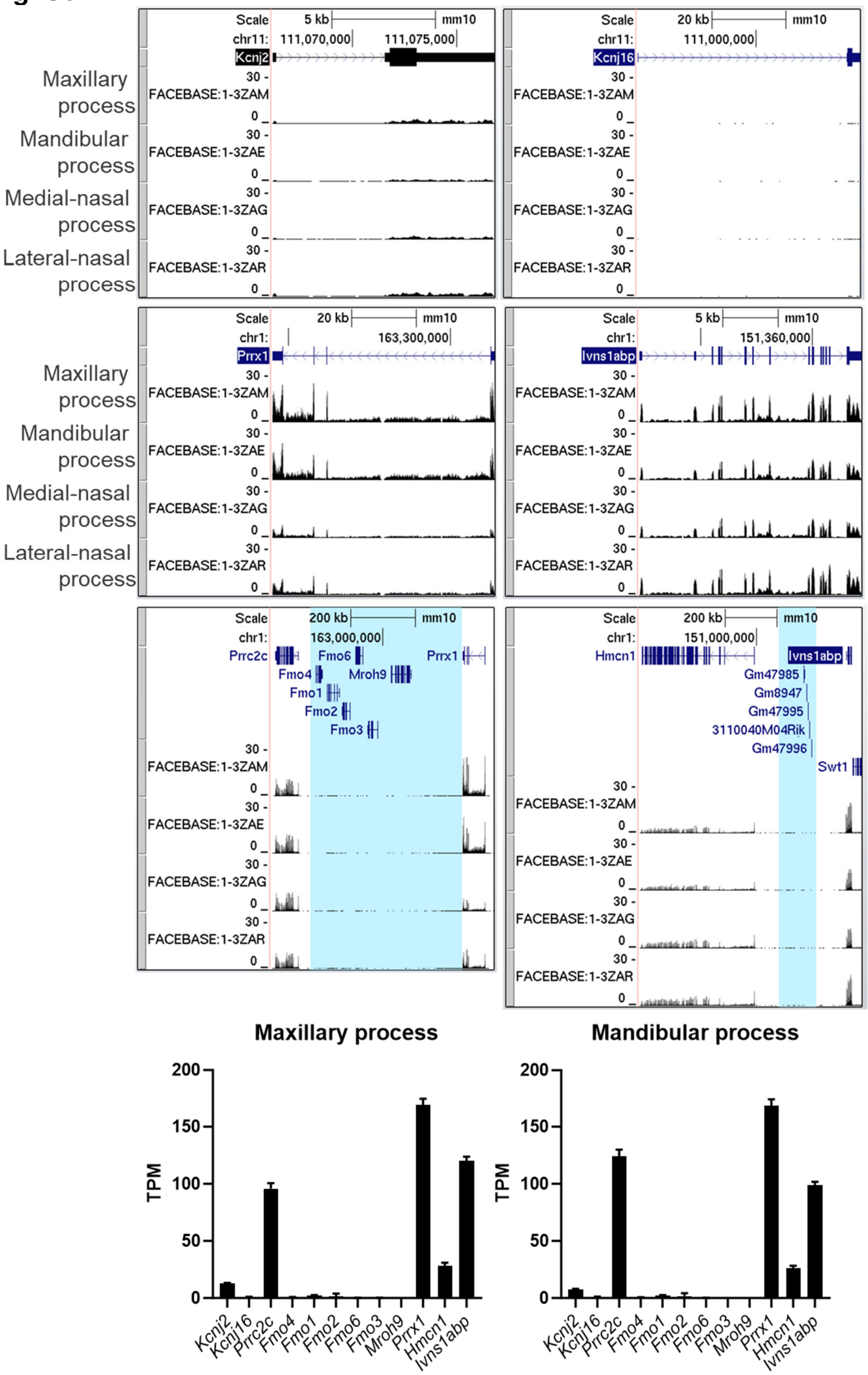


**Fig. S6.** UCSC tracks showing RNA-seq data from e11.5 mouse face subregions of *Kcnj2* and *Kcnj16* genes, and the flanking genes of the equivalent chr1_D and chr1_H (highlighted in light blue) in the mouse genome (mm10). Below, RNA-seq data is shown as TPM (transcripts per million) for the same genes, as well as for the genes included in the equivalent chr1_D for the maxillary and mandibular processes.

## Fig. S7

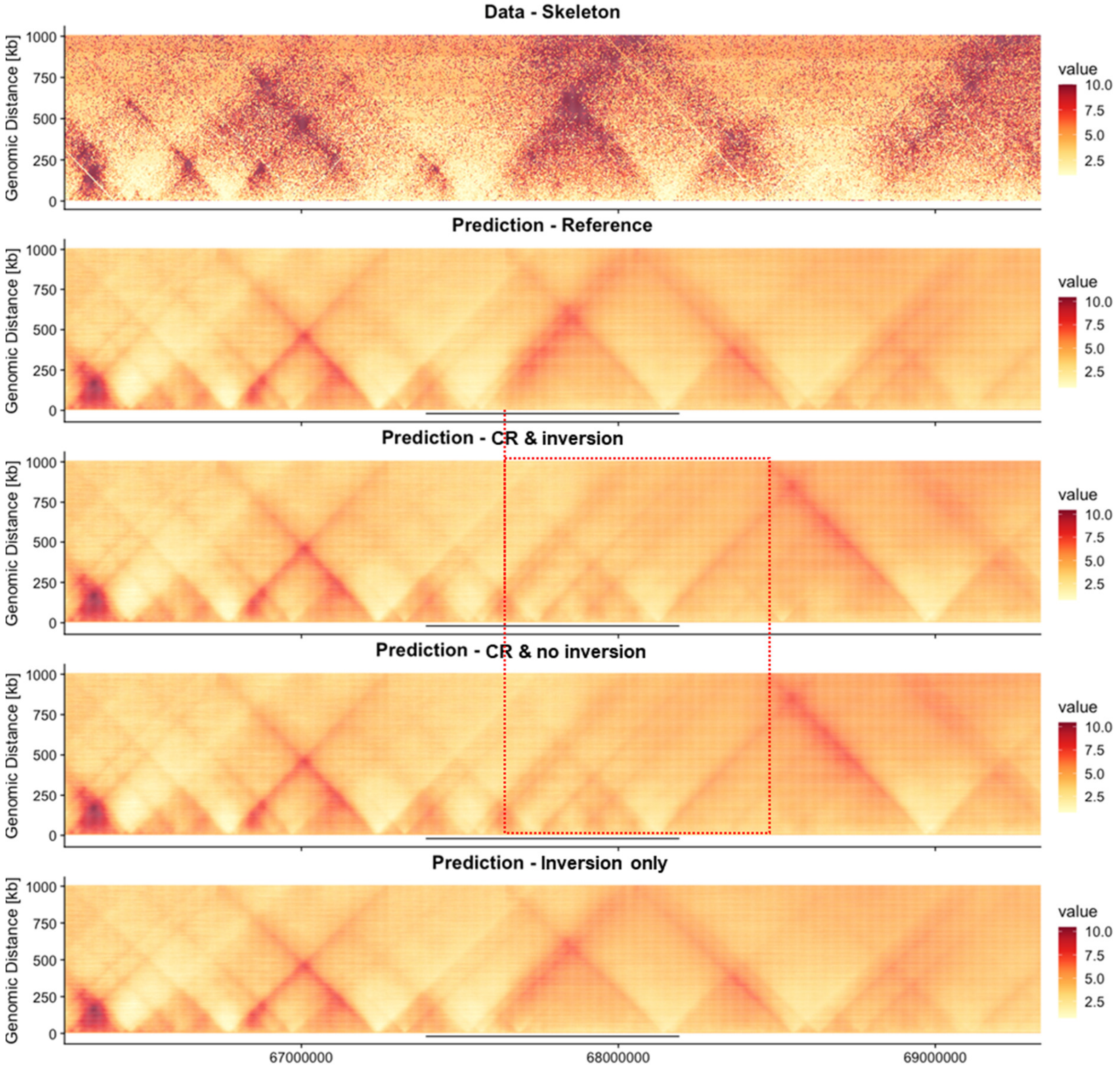


**Fig. S7.** Hi-C data and deepC predictions at the *KCNJ2-KCNJ16 locus*. At the top, the distance normalized Hi-C data (skeleton) of IMR90 cells is shown for the region of interest at chr17, whereas below, deepC predictions are shown for the reference sequence, the CR-containing equivalent variant (CR and inversion), the CR without the concomitant inversion (the position of the inserted CR from chr1 is indicated by the red box), and the inversion only, respectively. The color-coded values represent the interaction frequency in normalized Hi-C and predictions.

## Fig. S8

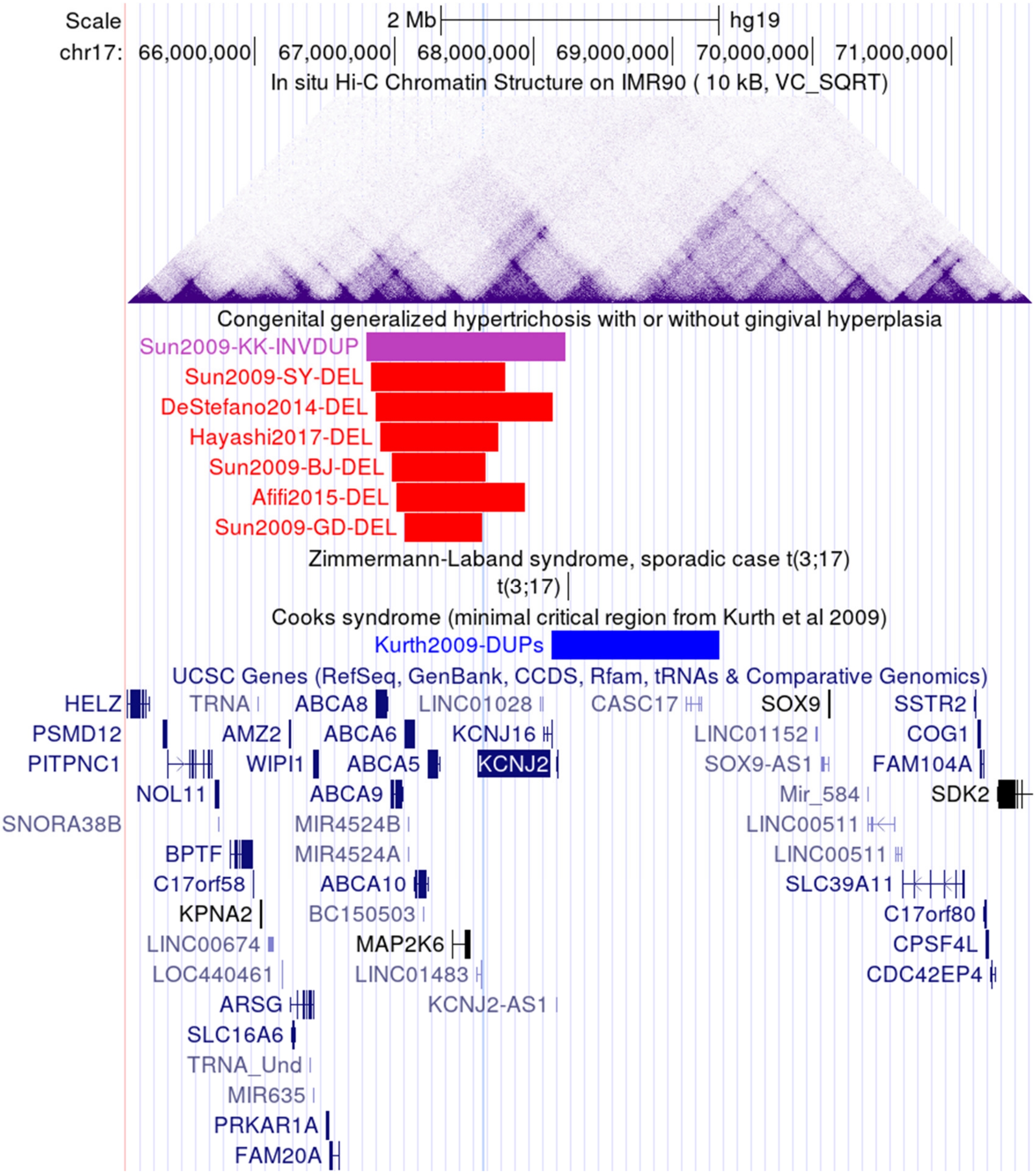


**Fig. S8.** TAD landscape and copy number and structural variants at human *ABCA-KCNJ locus*. UCSC tracks showing Hi-C data at the top, with the coordinates of the reported microdeletions (red) and the inverted duplication (purple) associated with congenital generalized hypertrichosis with or without gingival hyperplasia in the middle[1-4]. Additionally, the breakpoint of a reciprocal translocation t(3;17)(p14.3;q24.3) reported in a sporadic case of Zimmermann-Laband syndrome (black) and the minimal critical region of duplications responsible for Cooks syndrome (blue) are indicated[5-7]. The insertion point of the CR identified in the family is indicated with a vertical light blue line. At the bottom, the names and positions of the genes in the region are shown. Note that the deletions include the TAD and boundaries located near *MAP2K6*, suggesting that a misregulation effect on *KCNJ2/KCNJ16* is plausible.

## Supplemental References


1. Afifi HH, Fukai R, Miyake N, Gamal El Din AA, Eid MM, Eid OM, Thomas MM, El-Badry TH, Tosson AM, Abdel-Salam GM, Matsumoto N. De Novo 17q24.2-q24.3 microdeletion presenting with generalized hypertrichosis terminalis, gingival fibromatous hyperplasia, and distinctive facial features. *Am J Med Genet A.* 2015;167A:2418-2424.
2. DeStefano GM, Kurban M, Anyane-Yeboa K, Dall'Armi C, Di Paolo G, Feenstra H, Silverberg N, Rohena L, Lopez-Cepeda LD, Jobanputra V, Fantauzzo KA, Kiuru M, Tadin-Strapps M, Sobrino A, Vitebsky A, Warburton D, Levy B, Salas-Alanis JC, Christiano AM. Mutations in the cholesterol transporter gene ABCA5 are associated with excessive hair overgrowth. *PLoS Genet.* 2014;10:e1004333.
3. Hayashi R, Yoshida K, Abe R, Niizeki H, Shimomura Y. First Japanese case of congenital generalized hypertrichosis with a copy number variation on chromosome 17q24. *J Dermatol Sci.* 2017;85:63-65.
4. Sun M, Li N, Dong W, Chen Z, Liu Q, Xu Y, He G, Shi Y, Li X, Hao J, Luo Y, Shang D, Lv D, Ma F, Zhang D, Hua R, Lu C, Wen Y, Cao L, Irvine AD, McLean WH, Dong Q, Wang MR, Yu J, He L, Lo WH, Zhang X. Copy-number mutations on chromosome 17q24.2-q24.3 in congenital generalized hypertrichosis terminalis with or without gingival hyperplasia. *Am J Hum Genet.* 2009;84:807-813.
5. Kurth I, Klopocki E, Stricker S, van Oosterwijk J, Vanek S, Altmann J, Santos HG, van Harssel JJ, de Ravel T, Wilkie AO, Gal A, Mundlos S. Duplications of noncoding elements 5' of SOX9 are associated with brachydactyly-anonychia. *Nat Genet.* 2009;41:862-863.
6. Abo-Dalo B, Kim HG, Roes M, Stefanova M, Higgins A, Shen Y, Mundlos S, Quade BJ, Gusella JF, Kutsche K. Extensive molecular genetic analysis of the 3p14.3 region in patients with Zimmermann-Laband syndrome. *Am J Med Genet A.* 2007;143A:2668-2674.
7. Kim HG, Higgins AW, Herrick SR, Kishikawa S, Nicholson L, Kutsche K, Ligon AH, Harris DJ, MacDonald ME, Bruns GA, Morton CC, Quade BJ, Gusella JF. Candidate loci for Zimmermann-Laband syndrome at 3p14.3. *Am J Med Genet A.* 2007;143A:107-111.
